# Aerosolized BC-819 Inhibits Primary but Not Secondary Lung Cancer Growth

**DOI:** 10.1371/journal.pone.0020760

**Published:** 2011-06-08

**Authors:** Günther Hasenpusch, Corinna Pfeifer, Manish Kumar Aneja, Kai Wagner, Dietrich Reinhardt, Michal Gilon, Patricia Ohana, Avraham Hochberg, Carsten Rudolph

**Affiliations:** 1 Department of Pediatrics, Ludwig-Maximilians-University, Munich, Germany; 2 Institute of Pathology, Ludwig-Maximilians-University, Munich, Germany; 3 Department of Biological Chemistry, Institute of Life Sciences, Hebrew University, Jerusalem, Israel; Ludwig-Maximilians-Universität München, Germany

## Abstract

Despite numerous efforts, drug based treatments for patients suffering from lung cancer remains poor. As a promising alternative, we investigated the therapeutic potential of BC-819 for the treatment of lung cancer in mouse tumor models. BC-819 is a novel plasmid DNA which encodes for the A-fragment of Diphtheria toxin and has previously been shown to successfully inhibit tumor growth in human clinical study of bladder carcinoma. In a first set of experiments, we examined *in vitro* efficacy of BC-819 in human lung cancer cell-lines NCI-H460, NCI-H358 and A549, which revealed >90% reduction of cell growth. *In vivo* efficacy was examined in an orthotopic mouse xenograft lung cancer model and in a lung metastasis model using luminescent A549-C8-luc adenocarcinoma cells. These cells resulted in peri- and intra-bronchiolar tumors upon intrabronchial application and parenchymal tumors upon intravenous injection, respectively. Mice suffering from these lung tumors were treated with BC-819, complexed to branched polyethylenimine (PEI) and aerosolized to the mice once per week for a period of 10 weeks. Using this regimen, growth of intrabronchially induced lung tumors was significantly inhibited (p = 0.01), whereas no effect could be observed in mice suffering from lung metastasis. In summary, we suggest that aerosolized PEI/BC-819 is capable of reducing growth only in tumors arising from the luminal part of the airways and are therefore directly accessible for inhaled BC-819.

## Introduction

According to epidemiologic studies, lung cancer remains the leading cause of cancer related deaths worldwide with only 15 percent of all patients in USA and 10 percent of all patients in Europe surviving more than five years after diagnosis of the disease [1]. This situation is mainly due to late diagnosis of lung tumors and lack of therapeutic efficacy of currently available anticancer drugs, especially in advanced stages of the disease when surgical ablation is not feasible or tumors reoccur after surgery.

In order to close this therapeutic gap, gene therapy was suggested as promising opportunity for interfering with critical molecular pathways of cancer cells [2]. Recent strategies attempted in clinical studies included induction of tumor cell apoptosis through the replacement of mutated p53 tumor suppressor gene, tumor cell suicide through induction of genes encoding for specific enzymes which are sensitive to otherwise benign agents and activation of the immune system with genes encoding for cytokines [3]. The majority of gene therapeutic approaches are based on viral vectors which are highly efficient but due to development of neutralizing antibodies, unfavorable for repeated application. Non-viral vectors, though suitable for repeated application are relatively less efficient, especially in the airways [4].

Due to safety issues, gene therapeutic agents are preferably locally administered in current clinical trials. Indeed local application of drugs has been shown to be advantageous in various lung diseases, such as asthma, COPD and recently in lung cancer [5]. In case of lung cancer for example, application of chemotherapeutic drugs provided higher pulmonary drug levels and additionally reduced systemic side effects [6]. Due to these favorable distribution properties, aerosols have been considered for the application of gene therapeutic agents. Plasmid DNA, complexed with cationic polymers, resulted in successful transfection of bronchial and alveolar epithelium with reporter genes [7], [8] and reduced tumor growth when a therapeutic plasmid coding for p53 was applied to lung tumor bearing mice [9].

Furthermore, it would be desirable to restrict the activity of the applied plasmid DNA to the lung tumor cells only, thus leaving the healthy tissue unaffected. In this context, plasmid BC-819 (also known as DTA-H19) could be an ideal candidate. Plasmid BC-819 encodes the diphteria toxin A fragment under the control of the H19-promoter. Expression of diphteria toxin A fragment inevitably destroys cells by immediate disruption of protein synthesis and the H19 promoter restricts its expression to tumor cells [10]. H19 is a paternally imprinted, maternally expressed, oncofetal gene that has no protein product [11]. It is expressed at substantial levels in several different human tumor types, but is only marginally expressed or completely absent in normal adult tissues [12]. Recent data suggested a role for H19 in promoting cancer progression, angiogenesis and metastasis [13].

Even though aerosolized gene therapy has been shown to be effective in mouse models of lung cancer, we questioned whether the localization of tumors may have an effect on the efficacy of the tumor specific BC-819 plasmid. For this reason, plasmid BC-819 was applied as an aerosol in two groups of mice suffering from lung tumors either induced by intrabronchial or intravenous injection of human lung cancer cells. To qualify and quantify tumor development *in vivo*, luminescent cells were used to generate lung tumors.

## Materials and Methods

### Chemicals

Branched polyethylenimine (PEI, average molecular weight 25 kDa) was obtained from Sigma-Aldrich (Deisenhofen, Germany). PEI was diluted in distilled water (water for injection, B. Braun Melsungen AG, Melsungen, Germany) and adjusted to pH 7 with HCl. D-Luciferin was obtained from Synchem (Felsberg/Altenburg, Germany).

### Plasmids

The plasmids pCluc and pUC21 were obtained from Plasmid Factory (Bielefeld, Germany). The plasmid pCluc codes for the reporter enzyme luciferase derived from *Photinus pyralis*. The plasmid pUC21 was used as negative control. The plasmid BC-819 encodes for the A fragment of diphtheria toxin (DT-A) under control of the H19 promotor and has already been described elsewhere [10].

### Cell lines

The cell line A549 was derived from a human lung tumor and histologically classified as an adenocarcinoma [14]. The cell lines NCI-H460 and NCI-H358 were isolated from human specimen as well and histologically classified as large cell undifferentiated carcinoma (NCI-H460) and bronchioloalveolar cell carcinoma (NCI-H358). A549-C8-luc cells are A549 cells, stably transfected with the gene coding for the reporter enzyme luciferase. All the cell lines were obtained from DSMZ (German Collection of Microorganisms and Cell Cultures, Braunschweig, Germany) except for A549-C8-luc which was purchased from Caliper Life Sciences (Alameda, California).

### Animals

Due to disparities in the animals' tumor susceptibility, different mouse strains had to be used for the induction of primary and secondary lung tumors, depending on the application route of the tumor cells. T-cell deficient nude mice were described as good donors for intrabronchially applied tumor cells recently [15] and therefore chosen for the induction of primary lung tumors. Successful generation of lung tumors through systemic administration of tumor cells however was reported to require donor animals with a high level of immune deficiency. For this reason T-, B- and natural killer cell deficient CB17.Cg-*Prkdc* ^scid^ *Lyst* ^bg^-mice were used for the secondary tumor model, just as described by previous authors [16].

Prior to the experiments animals were acclimatized for at least 7 days. All further procedures were approved and controlled by the local ethics committee (Regierung von Oberbayern) under the approval number 16-08 and were conducted according to the guidelines of the German law of protection of animal life.

### Orthotopic tumor implantation procedure

#### Intrabronchial application

NMRI-*Foxn1^nu^* mice were anesthetized through intraperitoneal injection of medetomidine (11.5 µg/kg), midazolam (115 µg/kg) and fentanyl (1.15 µg/kg) and subsequently placed on an angular board in order to visualize the glottis using a light source, an otoscope and a modified spatula (Hallowell, USA). 1×10^6^ A549-luc-C8 cells diluted in 100 µl PBS were applied orotracheally with a blunt ended, bended 27 G stainless steel needle which was adapted to a 1 ml *Injekt-F* plastic syringe (B. Braun Melsungen AG, Melsungen, Germany). Subsequent to the implantation procedure, anesthesia was antagonized through the subcutaneous injection of an antidote consisting of atipamezol (50 µg/kg), flumazenil (10 µg/kg) and naloxon (24 µg/kg). Mice recovered from anesthesia within 15 min.

#### Intravenous application

Conscious CB17.Cg-*Prkdc* ^scid^ *Lyst* ^bg^-mice were placed in a mouse-restrainer (Braintree Scientific Inc, USA) and 1×10^6^ A549-C8-luc cells diluted in 100 µl PBS were injected directly into the dorsal tail-vein using a 29G U-100 Insulin syringe (BD Consumer Healthcare, Le Pont de Claix, France).

### In vivo imaging

Animals were anesthetized just as described before. D-luciferin substrate (3 mg/100 µl PBS per mouse) was applied by intraperitoneal injection in the group of mice suffering from lung tumors which were provoked by intravenous injection of A549-C8-luc cells. Mice suffering from tumors which were induced by intrabronchial application of A549-C8-luc received the same amount of D-Luciferin diluted in 50 µl PBS via the intranasal route [17]. Bioluminescence was measured 10 minutes later, using an IVIS 100 Imaging System (Xenogen, Alameda, USA) and the camera settings: field of view 10, f1 f-stop, high-resolution binning and exposure-time of 5 min. The signal was quantified and analyzed using the Living Image Software version 2.50 (Xenogen, Alameda, USA).

### Preparation of PEI-pDNA polyplexes

Branched polyethylenimine (PEI; average MW = 25 kDa) was obtained from Sigma-Aldrich (Deisenhofen, Germany), dissolved in water, and adjusted to pH 7 using HCl. Polyplexes for aerosol application were formulated as previously described [7]. Briefly: BC-819 plasmid and branched PEI were separately diluted in distilled water to 4 ml, resulting in concentrations of 0.25 mg/ml BC-819 and 0.33 mg/ml branched PEI, respectively (corresponding to a N/P ratio of 10). The pDNA solution was pipetted into the PEI solution and mixed by pipetting up and down to yield a final pDNA concentration of 0.125 mg/ml. The complexes were incubated for 20 min at ambient temperature before use.

### Aerosol application of PEI-pDNA polyplexes

Aerosol application was performed as described previously [18]. Briefly, a LC STAR Nebulizer (Pari, Starnberg, Germany), driven by high pressure, which was generated from a BOY®SX compressor (Pari, Starnberg, Germany) was connected to a spacer, filled with 500 g Drying pearls Orange (Sigma-Aldrich, Deisenhofen, Germany). The spacer in turn was connected to a plastic box with four small holes at the opposite side, in order to allow aerosol flow. In order to facilitate higher aerosol deposition in the lung, animals were encouraged for accelerated breathing by driving the compressor with synthetic air, enriched with 5% CO_2_.

### Histology

Animals were euthanized by intraperitoneal injection of pentobarbital. Subsequently the lungs were removed, inflated with 4% paraformaldehyde through the trachea and further processed for Haematoxylin-Eosin staining according to a standard protocol.

### In-situ hybridization

In-situ hybridization (ISH) was performed as described by Ariel et al [16], using the digoxigenin labelled H19 RNA probe or by a modification of this method using H19-LNA (locked nucleic acid) Dig-labelled probe synthesized at Exiqon.

### Statistics

Results are reported as means and standard deviations. Statistical significance between two groups was determined with a Mann-Whitney-U-Test for *in-vitro* results and with a Logrank-test for *in-vivo* results. Probability (P)<0.05 was considered significant. All statistical analyses were performed using the program StatView 5.0 (SAS Institute, Inc., Cary, NC, USA).

## Results

### Plasmid pBC-819 inhibits *in vitro* cell proliferation of human lung cancer cells

*In vitro* transfection experiments were conducted in different human lung cancer derived cell-lines. Increasing amounts of pBC-819 (0, 350 and 700 ng) were co-transfected with 100 ng of luciferase encoding plasmid. The total amount of pDNA was filled up to 800 ng for all the compared samples using pUC21 plasmid as filler pDNA. Hence, the efficacy of pBC-819 was indirectly determined by a reduction of luciferase activity, indicating destruction of transfected tumor cells.

**Figure 1:**
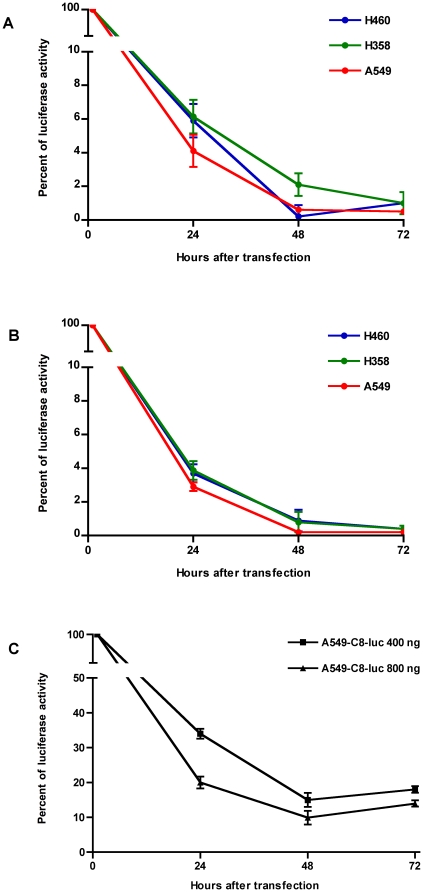
Effect of BC-819 on cell growth in different lung cancer cell lines. NCI-H460, NCI-H358 and A549 cells were transfected, using Lipofectamine 2000, with BC-819 and co-transfected with a plasmid, encoding for the reporter enzyme luciferase. As early as 24 h after transfection, luciferase activity (indirectly indicating cell growth) was reduced by at least >90% when 350 ng BC-819 was used (A). 48 hours later luciferase activity was decreased by more than 98%, except for the cell line NCI-H358. However, a luciferase decrease of more than 98% was observed in all cell lines when the amount of BC-819 was increased to 700 ng (B). The influence of BC-819 on bioluminescent A549-C8-luc was examined as well and revealed decreased luciferase and therefore reduced cell growth (more than 50%) as early as after 24 hours (C). Maximum inhibition of cell growth was reached by 48 hours after transfection, becoming apparent through more than 75% reduction in luciferase activity.

Using Lipofectamine 2000 as transfection reagent, a time and dose dependent decline of luciferase activity was observed in each of the cell lines used. The effect of pBC-819 became apparent as early as 24 hours after transfection with a reduction in luciferase activity of more than 90% when 350 ng (Fig. 1A) and more than 95% (Fig. 1B) when 700 ng of BC-819 were used. More than 98% of the luciferase activity was inhibited 48 hours after transfection when 700 ng BC-819 were used. Similar result was also obtained with 350 ng, except for the cell line NCI-H358.

These studies inspired us to screen this plasmid with the cell line A549-C8-luc, which had been stably transfected with luciferase. Measurement of luciferase at different time points post transfection showed that BC-819 was effective in A549-C8-luc cells as early as 24 hours after transfection (Fig. 1C). The lower concentration (400 ng) of BC-819 showed to be capable of reducing luciferase activity by more than 50%. Using 800 ng BC-819, it was possible to decrease the luciferase activity by more than 75%. Taken together these studies revealed a clear effect of plasmid BC-819 on cell viability of human lung cancer cells *in vitro*.

### Growth of lung tumors depends on the application site of tumor cells

**Figure 2:**
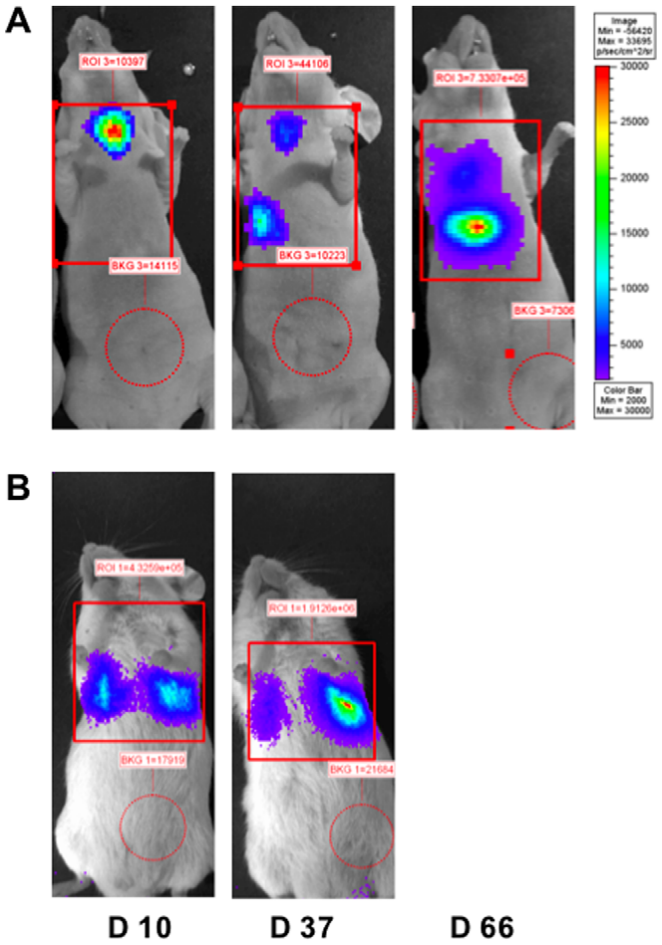
Bioluminescence imaging of lung tumors. Growth of orthotopic lung tumors was measured at defined time-points using bioluminescence imaging. Intrabronchially induced tumors (A) appeared initially in the jugular region and disseminated to the deeper lungs at later time points, whereas intravenously induced tumors (B) were restricted to the lung region only.

To evaluate the efficiency of pBC-819 aerosol therapy *in vivo*, orthotopic lung tumors were generated in immune deficient mice by intrabronchial or intravenous application of A549-C8-luc cells. We found that the choice of the application route largely affected the growth patterns of the resulting lung tumors and influenced animal survival. Intrabronchial application of A549-C8-luc cells gave rise to luminescent tumors, initially localized in the jugular region, by 10 days post transplantation and then disseminating caudally to the left or right lung within 66 days (Fig. 2A). However, tumors which were induced through intravenous injection of A549-C8-luc tumor cells, were characterized by disseminated bioluminescence signals in both lungs after 10 days which then continuously increased (Fig. 2B).

**Figure 3:**
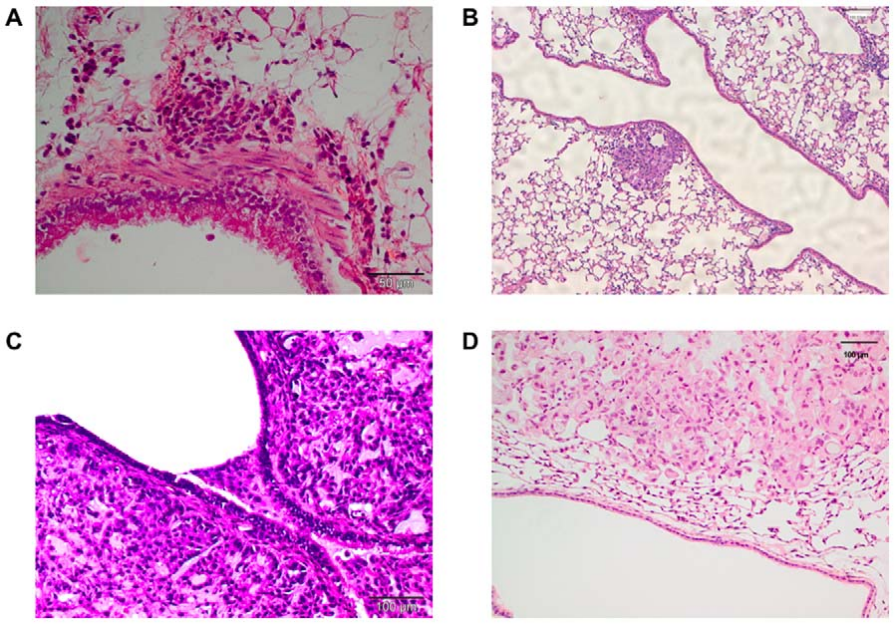
Histology of orthotopically induced lung tumors. Tumors were detectable as early as 10 days after intrabronchial implantation in the peribronchiolar region of the lungs (A). End stages of intrabronchially induced lung tumors were characterized by multiple, peribronchiolar and parenchymal localized tumors (B) and by tumor tissue within the luminal lung regions as well (C). Intravenous injection of A549-C8-luc, however, resulted in tumors predominately growing in the parenchyma of the lungs but not peribronchiolar or within the bronchi (D). Figure D shows parenchymal tumor tissue close to a bronchus, but not peribronchiolar. Alveolar tissue localized between the tumor and the bronchus however is condensed by the adjacent tumor nodule.

Histopathological post mortem examination of intrabronchially induced lung tumors revealed first evidence of tumor tissue within the peribronchiolar region after 10 days (Fig. 3A). Examinations at later time points revealed multiple tumor nodules, localized in the parenchyma, the peribronchiolar region and within the bronchia (Fig. 3 B–C). Lungs, affected from intravenously induced tumors, however, were characterized by disseminated tumor tissue only in the parenchyma of the organ (Fig. 3 D).

### A549-C8-luc based lung tumors are strongly positive for the H19 oncogene

**Figure 4:**
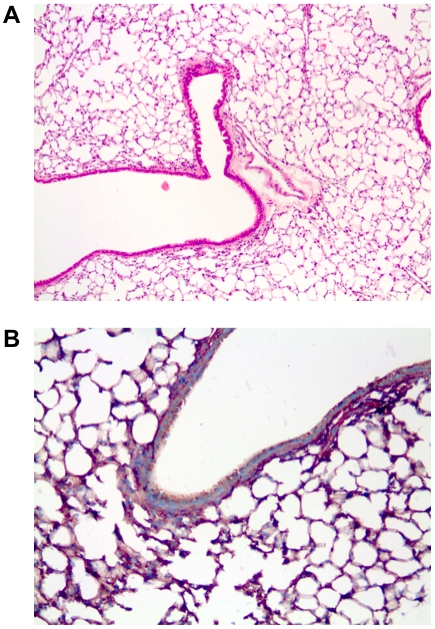
In-situ-hybridization for H19-activity in lung tumors. An intrabronchially induced lung tumor was stained for H.E. (A) and screened for H19-activity using ISH (B). In the given sample a strong expression of H19 was found, indicated by a brownish colorization of the examined tissue.

Because the presence of the non-coding RNA H19 is crucial for the efficacy of BC-819, we examined tumor tissue for the presence of non-coding H19 RNA. In-situ-hybridization revealed a strong expression of H19 in A549-C8-luc based lung tumors, indicated by brownish colorization of the examined tissue (Fig. 4).

### Inhalation of PEI/pBC-819 aerosol inhibits primary but not secondary tumor growth

Based on the results from *in vitro* experiments, the efficacy of pBC-819 was investigated *in vivo* through the application of aerosolized pBC-819 plasmid to lung tumor bearing mice. These experiments were conducted in mice suffering from either intrabronchially or intravenously induced lung tumors in order to mimic pBC-819's impact on primary and secondary lung tumors.

For subsequent aerosol application pBC-819 was complexed to branched PEI and administered in a modified whole-body inhalation chamber. Treatment of lung tumor bearing mice was started at 21 days after tumor cell transplantation, as soon as the evidence of lung tumors had been verified using *in vivo* bioluminescent imaging. The treatment protocol included one inhalation procedure a week for a total time period of 10 weeks.

**Figure 5:**
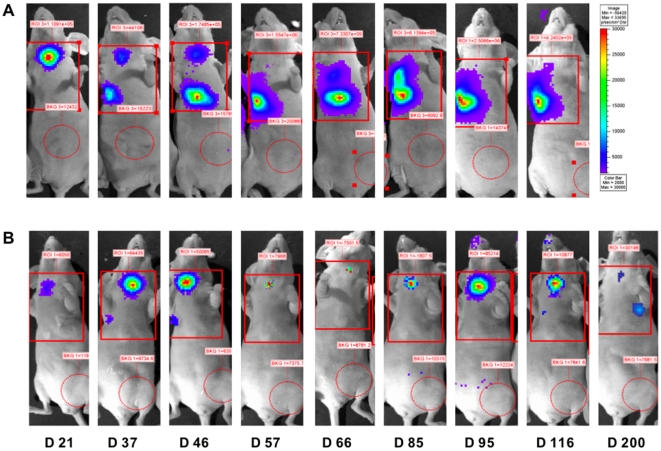
BLI of untreated and treated tumor-bearing mice at different time points. Growth of lung tumors in untreated mice was characterized by initial tumor appearance in the neck region and subsequently continuous dissemination into the lungs (A). This growth pattern differed in mice which were treated with BC-819 where tumors either decreased in size or in completely vanished during treatment.

These experiments revealed either completely abolished or severly attenuated tumor progression in mice suffering from intrabronchially induced lung tumors when treated with PEI/pBC-819 aerosol (Fig. 5A–B). A bioluminescence activity of more than 2×10^5^ photons per second was associated with a rapid decline of the animal's general condition (indicated by rapid weight loss and dyspnea) and therefore defined as endpoint-criteria. Based on this procedural method, a median survival of 66 days was calculated for animals of the untreated control group. In contrast to this, treated mice survived more than 200 days, even though aerosol application of PEI/pBC-819 aerosol was terminated more than 100 days earlier, on day 91. Except for one mouse, all treated mice (n = 5) survived more than 200 days, being either free of lung tumors or showing reduction of tumor growth and no impairment of the general condition. One mouse had no signs of tumor evidence in the lungs on day 200, but indeed one distant metastasis in the brain (data not shown).

**Figure 6:**
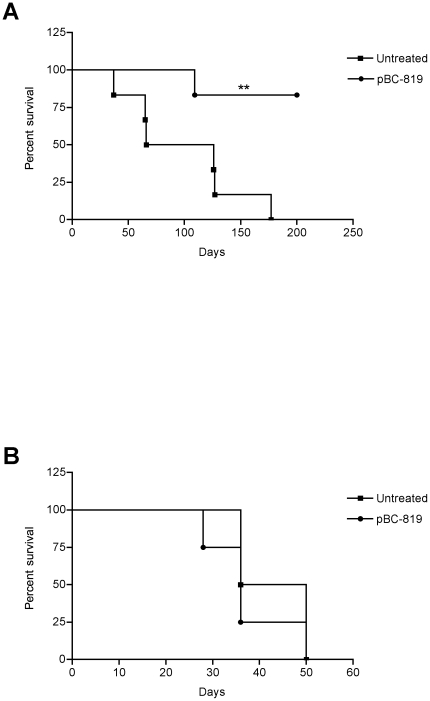
Survival of untreated and treated tumor-bearing mice. Mice bearing intrabronchially induced lung tumors showed a significant survival benefit (p = 0.01) when treated with BC-819 compared to animals which received no treatment (A). No survival benefit was observed in mice suffering from intravenously induced lung tumors, independent of treatment with BC-819 (B).

Finally, day 200 was elected to be the endpoint of the study. Statistical analysis of the results revealed a highly statistically significant difference (p = 0.01) between treatment and control group regarding the time which was necessary to reach the endpoint of the study (Fig. 6A).

Treatment with PEI/pBC-819-aerosol showed to be less efficient in mice suffering from lung tumors, which were generated through the intravenous injection of A549-C8-luc tumor cells. As mentioned before, tumor cells entered the lungs through blood circulation, consequently influencing localization and growth patterns of the lung tumors, resulting in tumors growing in the parenchyma, but not in the bronchi. Under this condition, treatment with PEI/pBC-819-aerosol resulted in no statistically significant difference between treated and untreated animals. Even though intravenously induced tumors grew more rapidly, impairment of the general condition was recognized as soon as a luciferase activity exceeded 1×10^6^ photons per second, which was therefore chosen as an endpoint for mice suffering from intravenously induced lung tumors. The median time to reach this endpoint was 43 days for the untreated animals and 36 days for the treated animals. Furthermore statistical analysis of these results revealed no significant difference (p = 0.3) between treated and untreated mice (Fig. 6B).

In summary, the results of these experiments demonstrate, that aerosolized PEI/pBC-819 is capable of inhibiting lung tumor growth in mice suffering from intrabronchially induced lung tumors. Moreover, the present results show that a comparable effect could not be observed in mice, which suffered from parenchymal lung tumors, after intravenous application of tumor cells.

## Discussion

Gene therapy is considered to facilitate treatment opportunities for incurable pulmonary diseases including monogenetic defects like cystic fibrosis, alpha_1_-antitrypsin deficiency and complex disease like asthma and lung cancer [19]. In this regard, local application of gene therapeutic drugs as aerosols has been considered to be advantageous for targeting of the lungs because i) the desired drug could be administered in a large quantity directly at the site of disease and ii) severe side effects which could be provoked by systemic application of nucleic acids and/or gene therapeutic vectors would be minimized. The concept of aerosol based gene therapy was further investigated in the present work through the application of the plasmid BC-819 in an orthotopic xenograft model of human lung cancer. In this regard, we investigated if tumor cell specific plasmids were capable of destroying only primary tumors of the luminal airways or if they were also capable of showing an effect against secondary tumors located in the parenchymal tissue. To address this question, lung tumors were induced using two different application routes of lung cancer cells. Tumors growing from the luminal side of the lungs were obtained when cells were applied intrabronchially and tumors growing from the blood vessels' side were obtained by applying the cells intravenously. Histology of intrabronchially induced tumors revealed evidence of tumors in the parenchyma of the organ and in the bronchial walls. These findings correspond with results, which were reported by other authors [15]. Intravenous injection of tumor cells, however, resulted in carcinomas only growing in the parenchyma of the lungs but not in the bronchial walls. Tumor growth monitoring using bioluminescent imaging furthered the results achieved from histology, revealing different growth patterns of tumors, which were induced intrabronchially and intravenously. Interestingly, we found that intrabronchially induced lung tumors arise from the peribronchiolar region which suggests that intrabronchially applied cells must be capable to invade and conquer the bronchial epithelia in order to colonize the peribronchiolar tissue. This finding is encouraging with respect to the results of other groups who demonstrated colonization of lung epithelia with labeled genetically engineered mesenchymal stem cells when applied through the intrabronchial route [20]. Based on the presumption that intrabronchially applied tumor cells initially invade the epithelial layers in the bronchial region to subsequently spread peribronchiolar, we suggested that treatment with aerosolized BC-819 should be efficient in destroying the tumor cells as we and others have previously shown that nebulized luciferase encoding plasmid DNA predominately transfects the bronchial and alveolar epithelia when complexed to branched PEI [7] [8].

In order to substantiate our assumption, we treated tumor-bearing mice with the plasmid BC-819 which has recently been shown to be therapeutically efficient in BCG-resistant bladder carcinomas in patients [21]. Plasmid BC-819 codes for the A fragment of diphtheria toxin and is driven by the H19 promotor. As H19 promoter is active only in malignant cells and during embryogenesis, tumor tissue is destroyed, while healthy tissue stays unharmed [10]. Prior to *in vivo* experiments the efficacy of BC-819 was determined in the lung cancer cell lines NCI-H460, NCI-H358 and A549 *in vitro*, revealing a considerably inhibiting effect on cell growth. It has been previously shown that the non-coding H19 mRNA is highly expressed, indicating active H19 promoter, in lung cancer cell lines, especially in A549 [22]. Consequently, lung cancers in mice were induced using the luciferase expressing cell line A549-C8-luc which had also been shown to be susceptible for the plasmid BC-819. Furthermore, the lungs were examined post mortem for H19-expression by In-situ-hybridization which revealed strong evidence of H19 expression in A549-C8-luc lung tumors.

This experiment revealed that weekly aerosol treatment with PEI/BC-819 gives a significant (p = 0.01) survival benefit for mice suffering from intrabronchially induced lung tumors but not to mice suffering from intravenously induced lung tumors (p = 0.36). Based on these results, we speculate that PEI/BC-819 aerosol treatment is only effective in primary lung tumors which are accessible through inhalation because the malignant growth predominately arises from the luminal part of the airways. In contrast to this, tumor growth of intravenously induced lung tumors primarily evolves from the blood vessels, subsequently invading the parenchyma of the lung. As mentioned before, aerosol based gene transfer is predominantly restricted to the bronchial epithelia. Certainly this does not mean that therapeutic effects could not be achieved for the entire organ. In fact several studies successfully demonstrated effects of nebulized pDNA in secondary models of lung cancer [23]. However, other plasmid constructs were used in these studies, without cancer cell specific promoters and including genes for less cell toxic proteins, e.g. the tumor suppressor protein p53. Furthermore, an apoptotic protein such as p53 will most likely not lead to immediate destruction of the transfected cell, therefore raising the possibility that excessive p53 could be released from the transfected cell or more likely, paracrine or interleukin mediated signaling could be activated in the surrounding tissue.

Even though aerosol application seems promising for future treatments of severe lung diseases, it should be kept in mind that this kind of therapy also seems much like a tightrope walk. Indeed, the likelihood of severe side effects, such as inflammation or allergic reactions, will increase with the toxicity and irritability of the applied substances, which consequently limits the advantage of the inhalation route. Concerns like these may have been considered in particular for the aerosol application of cytotoxic substances such as paclitaxel [6] or doxorubicin [5] but may also be relevant for the application of nucleic acids. Indeed a potent immune system not only acts on foreign pathogens but also on non-viral vectors and nucleic acids. However, only a slight increase of cytokines has been described for the aerosol application of branched PEI to the lungs [24], [25], [26]. Besides inflammation due to immune reactions, severe side effects could evolve from destroyed tissue. In this regard the particular architecture of the lungs should be considered. The high incidence of blood vessels raises the susceptibility for life threatening lung bleedings (hemoptysis), which could be induced by cell toxic substances [27]. Under this consideration it seems likely that tumor cell specific plasmids such as BC-819 which only destroys tumor but not healthy tissue could bring promising benefits for the aerosol treatment of lung cancers.
